# Variations in Cardiac Arrest Regionalization in California

**DOI:** 10.5811/westjem.2017.10.34869

**Published:** 2018-02-19

**Authors:** Brian L. Chang, Mary P. Mercer, Nichole Bosson, Karl A. Sporer

**Affiliations:** * University of California San Francisco School of Medicine, Department of Emergency Medicine, San Francisco, California; † Los Angeles County Emergency Medical Service Agency, Los Angeles, California; ‡ Harbor-UCLA Medical Center and the Los Angeles Biomedical Research Institute, Carson, California; § Alameda County Emergency Medical Service Agency, Alameda, California

## Abstract

**Introduction:**

The development of cardiac arrest centers and regionalization of systems of care may improve survival of patients with out-of-hospital cardiac arrest (OHCA). This survey of the local EMS agencies (LEMSA) in California was intended to determine current practices regarding the treatment and routing of OHCA patients and the extent to which EMS systems have regionalized OHCA care across California.

**Methods:**

We surveyed all of the 33 LEMSA in California regarding the treatment and routing of OHCA patients according to the current recommendations for OHCA management.

**Results:**

Two counties, representing 29% of the California population, have formally regionalized cardiac arrest care. Twenty of the remaining LEMSA have specific regionalization protocols to direct all OHCA patients with return of spontaneous circulation to designated percutaneous coronary intervention (PCI)-capable hospitals, representing another 36% of the population. There is large variation in LEMSA ability to influence inhospital care. Only 14 agencies (36%), representing 44% of the population, have access to hospital outcome data, including survival to hospital discharge and cerebral performance category scores.

**Conclusion:**

Regionalized care of OHCA is established in two of 33 California LEMSA, providing access to approximately one-third of California residents. Many other LEMSA direct OHCA patients to PCI-capable hospitals for primary PCI and targeted temperature management, but there is limited regional coordination and system quality improvement. Only one-third of LEMSA have access to hospital data for patient outcomes.

## INTRODUCTION

Annually over 400,000 people suffer non-traumatic out-of-hospital cardiac arrest (OHCA) in the United States.^1^,^2^ This represents the third leading cause of death in industrial nations and accounts for eight times as many deaths as motor vehicle collisions.^3^,^4^ There have been steady, albeit modest, improvements in the survival of patients with OHCA over the past decade.^5^ With recent advances in post cardiac arrest care, the proportion of patients who survive to hospital discharge after cardiopulmonary resuscitation (CPR) and return of spontaneous circulation (ROSC) has increased from one-third to one-half.^6^ Other improvements including higher rates of bystander CPR, dispatch-directed CPR, deployment of automatic external defibrillators in the community, and improved CPR quality have also contributed to increasing survival rates.^5^,^7^

Recently the American Heart Association (AHA) and other subject matter experts have advocated for the development of regional systems of cardiac arrest care with designation of cardiac arrest centers.^3^,^8^–^10^ A cardiac arrest center is a hospital that provides evidence-based practice in resuscitation and post-resuscitation care, including 24/7 percutaneous coronary intervention (PCI) capability and targeted temperature management (TTM), as well as an adequate annual volume of OHCA cases and a commitment to performance improvement and benchmarking. There is a similar precedent in the establishment of ST-segment elevation myocardial infarction (STEMI) centers over the past decade to improve outcomes in that time-dependent disease.^11^,^12^

Observational studies suggest a benefit of regionalization; therefore, the establishment of regional care systems may optimize access to and delivery of care for patients with OHCA. A prospective study demonstrated improved outcomes in patients with OHCA transported to a cardiac arrest center compared to non-cardiac arrest centers.^13^ There have been numerous observational studies with differing hospital characteristics^14^–^26^ as well as a number of studies that compared outcomes before and after the implementation of regionalized systems of care,^27^–^32^ all suggesting an association between improved survival and routing of select patients to cardiac arrest centers.

A regionalized cardiac arrest system involves a systematic approach to the care of the OHCA patients across a geographic area. This would include consistency in prehospital care, selective transport to designated cardiac arrest centers, consistent policies on the post-resuscitation care, and participation in a regional performance improvement process to address any potential disparities in care. Currently, most cardiac arrest centers in the U.S. are self-designated academic centers.^9^ The extent to which regionalization of cardiac arrest care has been established is not well quantified. Two studies describing established regional cardiac arrest care systems demonstrated improved patient outcomes with regionalization.^27^,^31^

This survey of local EMS agencies (LEMSA) in California was intended to determine the current practices regarding the treatment and routing of OHCA patients and the extent to which EMS systems have regionalized care across California.

## METHODS

The State of California has a population of 39 million, and EMS care is regulated by the California EMS Authority. Oversight of local care is provided by 33 LEMSA. These government agencies establish uniform policies and procedures for a countywide or region-wide (comprising multiple counties) system of first responders and EMS providers. While all LEMSA must have an EMS plan that conforms to California EMS Authority mandates, policies and protocols vary among them.^33^,^34^

We surveyed all 33 California LEMSA on three topics: 1) local policy regarding routing of OHCA patients to designated cardiac arrest centers; 2) specific interventions for post-resuscitation care available in those centers; and 3) access to data on OHCA treatment and outcome measures. We also requested system metrics on frequency of OHCA and patient outcomes. Of note, our survey inquired about the policies and protocols pertaining to all OHCA patients, not only those who achieved ROSC. We developed a 37-question survey (Appendix) in three sections: field treatment and routing policies (multiple choice); specialty centers (multiple choice); and system data (free response). Prior to dissemination, the survey was reviewed by several LEMSA administrators and subsequently edited for clarity. The survey was distributed by email to the California LEMSA administrators and medical directors in August 2016, available online via Qualtrics software. Reminders were sent until all LEMSA completed the survey. We clarified incomplete or inconsistent survey responses by email and/or phone.

**Population Health Research Capsule**

What do we already know about this issue?

Out-of-hospital cardiac arrest (OHCA) patients have better outcomes at cardiac arrest centers, and some emergency medical service (EMS) systems direct OHCA patients to such centers.

What was the research question?

How do EMS agencies in California route OHCA patients? What quality improvement metrics do they track?

What was the major finding of the study?

There is wide variation in the treatment and routing of OHCA patients in California. Only two of 33 EMS agencies have formal regionalized systems.

How does this improve population health?

This study suggests that EMS agencies in California can expand regionalized care for OHCA patients and increase quality improvement around OHCA systems.

The primary objective was to describe management of OHCA throughout California in terms of current treatment guidelines and specifically to determine the extent to which systems have regionalized care. Responses were submitted by either the LEMSA director or representative and downloaded or input into Excel (Microsoft Corporation, Redmond WA) for analysis. The findings of this study will be shared with the EMS Medical Directors Association of California (EMDAC), an advisory body to the California EMS Authority comprised of all EMS medical directors of the 33 LEMSAs, who meet quarterly to advise the state on issues pertaining to prehospital scope of practice and quality of care.

This study was submitted to the Institutional Review Board (IRB) at the University of California at San Francisco and was deemed to not involve human subjects as to require continuous IRB review.

## RESULTS

All 33 California LEMSA participated in the survey for a response rate of 100%. Table 1 provides a summary of LEMSA routing policies. Two LEMSA reported a fully developed regional cardiac arrest care system with specific clinical protocols to direct patients to cardiac arrest centers, a role in influencing hospital policies about post-cardiac arrest care, and participate in a regional performance-improvement process. The Los Angeles (LA) regional cardiac arrest system (population 10 million) has been described previously.^27^ In LA, all OHCA with ROSC and those transported with presumed cardiac etiology are routed to designated centers, which double as STEMI and cardiac arrest centers. All have 24/7 PCI capability, written internal protocols for TTM, and take part in a regional performance-improvement process. Alameda County (population 1.5 million) operates a similar system, routing all OHCA patients with ROSC at any time to cardiac arrest centers.

A large number of LEMSA (20/33), comprising a population of 14 million, have specific protocols to direct all OHCA patients with ROSC to designated PCI-capable hospitals. They have a limited role or no role in influencing hospital policies about post cardiac arrest care and do not have a regional performance-improvement process. There was inconsistency among agencies regarding the protocols and reporting required from these hospitals. Nearly all LEMSA (31/33) have a termination of resuscitation protocol for OHCA.

Eight LEMSA have policies and protocols that direct the use of TTM during post-resuscitation care, requiring hospitals to have a written TTM protocol, and five have a memorandum of understanding to enforce the requirement and allow them a role in determining the inclusion and exclusion criteria. Six LEMSA have protocols for the prehospital administration of therapeutic hypothermia.

Seven LEMSA have policies that require receiving hospitals to have a written protocol for emergent PCI after OHCA. Of these, four have memoranda of understanding with the hospitals and three have a role in determining inclusion and exclusion criteria. The use of PCI for patients with persistent cardiac arrest was rare. Fifteen agencies reported that this occurred in their system, but none reported more than 3–5 patients. Eleven LEMSA have hospitals with extracorporeal membrane oxygenation (ECMO) capability, but it was rarely used for this indication and there were no LEMSA with specific routing or regional policies for its use. Mechanical CPR devices were optional for 18 local EMS agencies. One agency required the use of mechanical CPR devices during transport and another required them for all OHCA patients.

The majority of LEMSA report collecting process measures for system quality improvement, with EMS response time the most commonly measured (30/33), followed by the time to CPR (24/33), the time to defibrillation (25/33), and the rate of dispatcher-assisted CPR (18/33). However, the measurements of in-hospital outcomes were significantly lower (14/33) with survival to hospital discharge the most commonly measured. The frequency of reported treatment and outcome measures are listed in Table 2.

## DISCUSSION

We present the current policies for treatment and routing of all OHCA patients throughout California with a 100% survey response rate. While the majority of LEMSA route cardiac arrest patients to specific specialty centers (PCI-capable hospitals), only two have formally regionalized cardiac arrest care systems, covering 39% of the California population. A surprising finding of our survey was the larger number of more informal designation of cardiac arrest centers without regionalization. A total of 20 out of 33 LEMSA (representing a population of 14 million or 36% of the population) had specific protocols to direct OCHA patients to STEMI-designated hospitals. A regionalized cardiac arrest system not only directs OHCA patients to designated hospitals, but also establishes a systemwide approach to cardiac arrest management and quality improvement to optimize resuscitation and post-resuscitation care. This is a multi-disciplinary approach that involves prehospital and inhospital care, including the appropriate and timely use of TTM and PCI, as well as consistent intensive care unit care and uniform prognostication.

Designated cardiac arrest centers that operate within a regionalized system with a robust performance-improvement process are likely to decrease the variability in care and improve outcomes. Several studies have demonstrated that survival after ROSC from an OHCA can vary considerably depending on the hospital and its clinical characteristics.^14^,^16^,^18^,^35^ In one Swedish study, the survival of OHCA patients with ROSC ranged from 14% to 42%.^20^ In 2008, Arizona designated 31 cardiac arrest centers and routed all OHCA patients to these centers. Before and after analyses of the Arizona Department of Health Services statewide, EMS cardiac-arrest database demonstrated increased neurologically intact survival with regionalization, as well as improved adherence to post-resuscitation care guidelines at designated centers.

However, routing of patients alone, even without the regionalized system infrastructure, may improve outcomes. As demonstrated in other complicated medical conditions, a number of studies have suggested that increasing the volume of OHCA patients receiving care at a particular hospital is associated with improved outcomes. Hospitals with high volumes of CPR cases demonstrate better outcomes for OHCA patients than those with lower volume, despite longer transport times to these cardiac arrest centers.^10^,^13^,^16^,^17^,^36^–^39^ Using California statewide data from 2011, we found that 10% of hospitals are defined as AHA Level I cardiac receiving centers, capable of providing 24-hour PCI and TTM, and meet a minimum volume of OHCA patients.^40^ As of 2011, these hospitals treated approximately 25% of the OHCA patients in California. The designation of cardiac arrest centers may be associated with an increased use of TTM and PCI and the rate of their use correlates with their survival rate with neurologically favorable outcome.^41^ While this is of particular interest to rural communities, where it may be advantageous for the OHCA patient to bypass the closest hospital or use air transportation to reach a cardiac arrest center, further work is necessary to determine what length-of-transport time and characteristics of cardiac arrest have maximal benefit in transport to a specialized center.

Still, quality improvement is essential to continue to improve system outcomes. A concerning finding of the survey was that only 14 LEMSA (42%) have access to the hospital outcomes. All successful systems that have improved their cardiac arrest survival required measuring different components of their system, with hospital outcomes one of the most important.^2^,^5^,^42^,^43^

These survey results provide an important foundation from which to move forward. There are a number of future opportunities for inquiry and improvement of cardiac arrest systems in California. As discussed above, the availability of hospital outcomes is essential for system quality improvement. The use of consistent definitions and inclusion/exclusion criteria developed by national or international organizations is also important to effectively benchmark. Given the low usage of ECMO or PCI during cardiac arrest, there may be opportunities to standardize their use across each region.^44^ The development of regional systems of care with designated cardiac arrest centers may allow for more rapid adoption of current and future evidence-based advances in care. There may also be a role for secondary transfer of OHCA patients with sustained ROSC who present to a non-cardiac arrest center, but this has yet to be established.^45^

## LIMITATIONS

Given the survey design, the study results are limited by self-reporting bias and potential misclassification bias by the survey participants. Question misinterpretation is possible; however, we attempted to mitigate this by careful review and follow-up with individual respondents for any discrepancies in responses. The survey only included California LEMSA; therefore, results may not be generalizable to other U.S. regions. Additionally, the survey did not ask explicitly about LEMSA participation in national, cardiac-arrest registry reporting, or collect numbers regarding OHCA patients and outcomes across all LEMSA. This study did not evaluate specific quality indicators or system performance in the individual EMS systems or differentiate between rural and urban LEMSA.

## CONCLUSION

Regionalized care of OHCA is established in two of 33 California LEMSA, providing access to approximately one-third of California residents. Many other LEMSA direct OHCA patients to PCI-capable hospitals for primary PCI and targeted temperature management, but there is limited regional coordination and system quality improvement. Only one-third of LEMSA have access to hospital data for patient outcomes.

## Supplementary Information



## Figures and Tables

**Table 1:** Summary of the routing and treatment policies for out-of-hospital cardiac arrest (OHCA) among the 33 local EMS agencies (LEMSA) in California.

	Number of LEMSAs in CA
Field treatment and routing policies	
Allow for the routing of OHCA to specific hospitals	23 (70%)
Require the routing of all OHCA to specific hospitals	5 (15%)
Have a Termination of Resuscitation policy for OHCA	31 (94%)
Route all persistent Vfib to specific hospitals	10 (30%)
Have a policy for pre-hospital initiation of Targeted Temperature Management (TTM)	6 (18%)
Require the use of mechanical CPR devices during transport	2 (6%)
Specialty centers	
Require that a written TTM policy exist at the receiving hospital	8 (24%)
Require that a written policy for emergent coronary angiography exist at the receiving hospital	8 (24%)
Require the transport of OHCA to a hospital with 24 hour capability for percutaneous coronary intervention	18 (55%)
Require the use of mechanical CPR devices in hospitals receiving OHCA	1 (3%)
Have hospitals that perform PCI for patients in persistent cardiac arrest	15 (45%)
Have hospitals that initiate ECMO for patients in persistent cardiac arrest	11 (33%)
Outcomes data	
Collect outcomes data on OHCA	26 (79%)

*CPR,* cardiopulmonary resuscitation; *PCI,* percutaneous coronary intervention; *ECMO,* extracorporeal membrane oxygenation; *Vfib,* ventricular fibrillation*.*

**Table 2:** Number of California local EMS Agencies (LEMSA) that track and maintain quality improvement processes and outcomes in cardiac-arrest care metrics.

	Pre-hospital outcomes				In hospital outcomes				
		
	EMS response time	Time to CPR	Time to defibrillation	Rate of dispatcher assisted CPR	Survival to hospital discharge	CPC or mRS scores at discharge	Risk-adjusted mortality	Frequency of TTM	Frequency of emergent coronary angiography
Yes	30 (91%)	24 (73%)	25 (76%)	18 (55%)	14 (42%)	10 (30%)	1 (3%)	3 (9%)	9 (27%)
Partially					4 (12%)	5 (15%)	1 (3%)	3 (9%)	5 (15%)
No	3 (9%)	9 (27%)	8 (24%)	15 (45%)	15 (45%)	18 (55%)	31 (94%)	27 (82%)	19 (58%)

*EMS*, emergency medical services; *CPR,* cardiopulmonary resuscitation; *CPC*, cerebral performance category; *mRS*, modified Rankin score; *TTM*, targeted temperature management; *PCI*, percutaneous coronary angiography
