# Supplementary material for: Variations in Cardiac Arrest Regionalization in California
Source: West J Emerg Med. 2018 Feb 19;19(2):259–65. doi: 10.5811/westjem.2017.10.34869 (PMC5851497; doi:10.5811/westjem.2017.10.34869)
Supplement: Supplementary file 1 [file wjem-19-259-s001.docx]

**Appendix.** Cardiac arrest center survey.

**Cardiac Arrest Center Survey**

This survey is to help understand how local EMS agencies in California are routing and managing cardiac arrest patients and how they are utilizing Cardiac Arrest Receiving Centers. 

You may add additional comments or elaborate on your response for any of the questions at the end of this survey.  You may contact the survey administrator (contact below) with questions while completing the survey.

We are sending this survey to the LEMSA Medical Directors. If you are NOT a LEMSA medical director (or someone who is designated to complete this survey) and believe you have received this email in error,  please do NOT complete this survey.

Only one survey is needed per LEMSA.

Please note, we have requested the contact information for the person completing this survey, in case we need to to clarify a response or comment.

Thank you for your time!

Brian Chang, MD

Mary Mercer, MD

Karl Sporer, MD

(contact: brian.chang@ucsf.edu)

**Field treatment and routing policies:**

1. Do your LEMSA policies allow routing of cardiac arrest patients to specific hospitals?

Yes (continue to question 1.a.

No (continue to question 2)

1.a. Are **ALL** cardiac arrest patients routed to specific hospitals?

Yes (continue to question 2)

No (continue to question 1.b.)

1.b. Does your LEMSA policy route to specific hospitals (select all that apply):

All ROSC?

Persistent VF?

Other? (Specify criteria)

1. Does your LEMSA have a Termination of Resuscitation (TOR) policy for out-of-hospital cardiac arrest (OHCA)?

Yes (continue to question 2.a.

No (continue to question 3)

2.a. Does your policy include base hospital consultation?

Yes (continue to question 2.b.

No (continue to question 3)

2.b. What are the cases that require consultation? (select one)

Some defined cases (please specify) ____________________________

For all terminations

1. Does your internal QI process for prehospital management of OHCA review the following: (check all that apply)

Response time of EMS provider

Time to CPR

Time to Defibrillation

Quality of CPR

Dispatcher assisted CPR

1. Does your LEMSA have a policy or protocol for initiation of Targeted Temperature Management (TTM) in the field?

Yes

No

1. Does your LEMSA’s OHCA policy require the use of mechanical CPR devices during patient transport?

Yes, required for all

No

Optional

**Specialty centers:**

1. Does your LEMSA require that a written Targeted Temperature Management (TTM) policy exist at the receiving hospital?

Yes (continue to 6.a, 6.b, 6.c)

No (continue to 7)

6.a. Is this requirement established by LEMSA policy?

Yes

No

6.b. Is this requirement established through a Memorandum of Understanding?

Yes

No

6.c. Does your LEMSA play a role in defining the inclusion and exclusion criteria for TTM?

Yes

No

1. Does your LEMSA require that a written policy for emergent coronary angiography of ROSC patients exist at the receiving hospital?

Yes (continue to 7.a, 7.b, 7.c)

No (continue to 7.d)

7.a. Is this requirement established by LEMSA policy?

Yes

No

7.b. Is this requirement established in a Memorandum of Understanding?

Yes

No

7.c. Does your LEMSA play a role in defining the inclusion and exclusion criteria for emergent coronary angiography of ROSC patients?

Yes

No

7.d. Does your LEMSA require transport to designated centers that have 24 hour capability for PCI?

Yes

No

1. Does your LEMSA require the use of mechanical CPR devices at hospitals?

Yes

No

1. Do any of your hospitals ever perform emergent coronary angiography and PCI on patients in persistent cardiac arrest?

Yes (continue to 9.a, 9.b)

No (continue to 10)

9.a. Specify the estimated number of patients per year ________

9.b. Are there specific criteria for coronary angiography in persistent cardiac arrest?

Yes (continue to 9.c)

No (continue to 10)

9.c. Does your LEMSA play a role in defining the inclusion and exclusion criteria for emergent coronary angiography in patients with persistent cardiac arrest?

Yes

No

1. Do any of your hospitals ever use extracorporeal membrane oxygenation (ECMO) for patients in persistent cardiac arrest?

Yes (continue to 10.a-d)

No (continue to 10.e)

10.a. Specify the estimated number of patients per year ____________

10.b. Are there specific criteria for ECMO in persistent cardiac arrest?

Yes (continue to 10.c)

No (continue to 11)

10.c. Does your LEMSA play a role in defining the inclusion and exclusion criteria for ECMO in patients with persistent cardiac arrest?

10.d. Is ECMO used for adult or pediatric patients or both?

Adult

Pediatric

10.e. Do any of your hospitals have a care and transfer agreement with an ECMO center for “ECMO to GO”?

Yes

No

**System data:**

1. Does your LEMSA collect metrics on out-of-hospital cardiac arrest (OHCA)?

Yes

No

1. Does your LEMSA designate specific hospitals as Cardiac Arrest Receiving Centers, and route non-traumatic OHCA to these hospitals?

Yes (continue to 13, 14, 15)

No (continue to 16)

1. (**if question 12 is yes**) Please specify designation year of first Cardiac Arrest Receiving Center: ______
2. (**if question 12 is yes**) Please provide the following numbers, or leave blank if this data was not collected. For year prior to designation, please provide data from the calendar year before designation of the first Cardiac Arrest Receiving Center. For example, if your first designation year was 2010, please provide data from 2009.

|  | 2015 | Year prior to designation |
| --- | --- | --- |
| OHCA who received active resuscitation (1) |  |  |
| OHCA transported to any hospital (2) |  |  |
| OHCA who achieved ROSC (3) |  |  |
| OHCA who survived to hospital admission (4) |  |  |
| OHCA who survived to hospital discharge (5) |  |  |
| OHCA who survived to hospital discharge with good neurologic status, with Cerebral Performance Category (CPC) of 1 or 2 (6) |  |  |
| OHCA who survived to hospital discharge with Modified Rankin Scale (mRS) of 0-2 (7) |  |  |

1. (**if question 12 is yes**) Please provide the following numbers, or leave blank if this data was not collected.

|  | 2015 |
| --- | --- |
| OHCA transported to designated Cardiac Arrest Receiving Centers (1) |  |
| OHCA who survived to hospital discharge at Cardiac Arrest Receiving Centers (2) |  |
| OHCA who survive to hospital discharge with good neurologic outcome (Cerebral Performance Category (CPC 1 or 2), Modified Rankin Scale (mRS) 0-2) at Cardiac Arrest Receiving Centers (3) |  |
| OHCA treated with TTM at Cardiac Arrest Receiving Centers (4) |  |
| OHCA who receive emergent coronary angiography at Cardiac Arrest Receiving Centers (5) |  |

1. (**if question 11 is yes**) Please provide the following numbers, or leave blank if this data was not collected.

|  | 2015 |
| --- | --- |
| OHCA who received active resuscitation (1) |  |
| OHCA transported to any hospital (2) |  |
| OHCA who achieved ROSC (3) |  |
| OHCA who survived to hospital admission (4) |  |
| OHCA who survived to hospital discharge (5) |  |
| OHCA who survived to hospital discharge with good neurologic status, with Cerebral Performance Category (CPC) of 1 or 2 (6) |  |
| OHCA who survived to hospital discharge with Modified Rankin Scale (mRS) of 0-2 (7) |  |

1. Does your internal QI process review the following by hospital?

|  |  | | | If Yes, what year did this start? |
| --- | --- | --- | --- | --- |
|  | Yes | Partially | No | Year |
| Survival to hospital discharge (1) |  |  |  |  |
| CPC or mRS scores at discharge (2) |  |  |  |  |
| Risk-adjusted mortality (3) |  |  |  |  |
| Frequency of TTM (4) |  |  |  |  |
| Frequency of emergent coronary angiography (5) |  |  |  |  |
| Frequency of PCI (6) |  |  |  |  |
